# Personality types and subjective well-being among people living with HIV: a latent profile analysis

**DOI:** 10.1007/s11136-019-02288-5

**Published:** 2019-09-10

**Authors:** Marcin Rzeszutek, Ewa Gruszczyńska

**Affiliations:** 1 Faculty of Psychology, University of Warsaw, Stawki 5/7, 00-183 Warsaw, Poland; 2 Faculty of Psychology, University of Social Sciences and Humanities, Chodakowska 19/31, 03-815 Warsaw, Poland

**Keywords:** HIV/AIDS, Personality types, Typological approach

## Abstract

**Purpose:**

We examined whether three types of personality (i.e. resilient, undercontrolled and overcontrolled) based on the Big Five personality taxonomy could be replicated among people living with HIV (PLWH). We also aimed to establish significant sociodemographic and clinical covariates of profile membership and verify whether these profiles are related to the subjective well-being (SWB) of participants.

**Methods:**

770 PLWH participated in this study. The Big Five personality traits were evaluated with the NEO-FFI questionnaire. SWB was operationalised by satisfaction with life (Satisfaction with Life Scale) and positive and negative affects (PANAS-X). Moreover, sociodemographic and clinical variables were collected.

**Results:**

Latent profile analysis was used to identify personality types among participants. Instead of the three profiles most frequently reported in the literature, we identified a four-profile model (the resilient, undercontrolled, overcontrolled and the average profile type) as the best fit to the data. These profiles did not differ with regard to sociodemographic and clinical covariates. However, significant differences in SWB across profiles were noted, i.e. the highest SWB was observed among members of the resilient profile, and overcontrollers and undercontrollers were almost equally regarded as second best in SWB level, whereas the average profile consists of PLWH with the worst SWB.

**Conclusion:**

Identifying personality types in clinical settings enables more comprehensive understanding of interrelations between personality and health. Regarding PLWH, the typological approach may shed new light on ambiguous results devoted to the role of personality in well-being of these patients.

## Introduction

For at least two decades, there has been ongoing debate between proponents of the traditional, dimensional approach to the study of personality traits [e.g. 1, 2] and their opponents, advocating for broader implementation of the typological perspective, which operationalises personality not via interindividual differences across isolated traits, but in terms of broader personality types that characterise each person [e.g. 3–5]. More specifically, the authors representing this latter standpoint argue that the dimensional approach is lacking in describing interrelated configurations within personality structure as well as their dynamics [6], which is one of the core elements across various personality definitions, starting even from the classic conceptualisations of this term [7]. The typological approach to personality is obviously not a new idea and has a long history in personality psychology, but it relied mainly on theoretically vague constructs devoid of empirical evidence [8]. However, over the last 20 years, several authors have built a solid empirical basis to understand personality as a functional whole, going beyond a set of separately analysed dimensions [9]. With regard to such understanding of personality, the most common typology was first provided by Robins et al. [4], who distinguished three personality types relying on ego-resiliency and ego-control theory (see [10]): in other words, resilient type (e.g. ‘self-confident, emotionally stable, energetic’), undercontrolled type (‘stubborn, active, impulsive’) and overcontrolled type (‘sensitive, introverted and dependable’). In the subsequent years, this typology was replicated in many samples with various methods and personality theories [e.g. 11, 12], including Big Five personality traits [3, 13].

As far as Big Five taxonomy is concerned, the resilient type is characterised by high extraversion and conscientiousness, low neuroticism and relatively high values on the other traits; the overcontrolled type reveals especially high neuroticism and conscientiousness, low extraversion and openness; and the undercontrolled type obtains predominantly low scores in conscientiousness and agreeableness [3, 14]. In addition, several studies found that overcontrolled and undercontrolled individuals have internalising problems (e.g. depression, anxiety, shyness and low sociability) and externalising problems (e.g., aggression, attention problems, but high sociability), respectively, whereas resilient people are usually free from both these tendencies [e.g. 9, 12].

Until now, almost all studies on personality types have been conducted in non-clinical samples [8, 9], thus very little is known whether these three types of personality are recognisable also in the clinical settings among individuals struggling with chronic disease and related psychological distress [15]. It is especially important regarding studies suggesting that personality is more strongly associated with subjective health indicators (e.g. distress, quality of life) compared with objective medical parameters [16]. Moreover, identifying personality types in clinical samples may shed new light on inconclusive findings on the link between personality, health and well-being [17]. For example, extraversion consists of two facets, both of which have contradictory effects on health outcomes. Namely, whereas positive affect is usually predictor of good health and reduced risk of illness [18], sensation seeking is mostly associated with risky health behaviours and substance use [19]. Furthermore, it was observed that extraversion might be differently linked to health depending on its link with other Big Five traits, particularly with conscientiousness [11].

The relationship between personality, health and well-being is of special importance with regard to people living with HIV (PLWH), who despite great progress in HIV treatment and increasing life expectancy [20], are still faced with intense HIV-related distress [21, 22]. Particularly, PLWH are constantly reporting lower levels of well-being, not only in comparison with the general population [23] but also against other chronic illnesses [24]. When looking for factors associated with well-being of these patients, an interesting trend emerges associated with the changing nature of this illness with time, from fatal disease in the past to manageable chronic health problem at present [25]. Namely, compared with older studies pointing to the major role of clinical variables [e.g. 26, 27], an increasing number of studies have recently reported that psychosocial factors outweighed the role of medical factors as predictors of PLWH well-being [e.g. 28, 29]. Out of these psychosocial factors, personality traits postulated by the Big Five theory may play a major role [30–32].

Some authors observed the differences between the selected Big Five traits (i.e. higher neuroticism and lower conscientiousness) among PLWH compared with the general US population [33]. It is still an open question whether the differences in personality may be the reason for undertaking risky health behaviours, as a potential pathway to HIV infection [34]. However, the above-mentioned studies were based on the traditional dimensional approach, and thus provided sometimes equivocal findings with respect to the relationship between personality and various dimensions of well-being [e.g. extraversion, 31 vs. 32]. Thus, implementing the typological approach may bring new understanding to the ambiguous results concerning the relationship of personality traits with various aspects of functioning of PLWH.

## Current study

In line with the reasoning set out above, the aim of our study was threefold. First, we wanted to verify whether the most often recognised three types of personality (i.e. resilient, undercontrolled and overcontrolled) could also be identified among the clinical sample of PLWH. Additionally, we investigated if there are differences in Big Five personality traits between PLWH and the Polish general population. Second, we examined which sociodemographic and clinical variables are significantly associated with the obtained personality types. Finally, we tested if these types of personality are related to the subjective well-being (i.e. satisfaction with life and positive and negative affects), after controlling for sociodemographic and clinical correlates.

## Method

### Participants and procedure

Participants were recruited from the State Hospital of infectious diseases outpatient clinic. The following eligibility criteria were implemented: 18 years of age or older, confirmed medical diagnosis of HIV+ and having received antiretroviral treatment in the clinic where the study was organised. The exclusion criteria were HIV-related cognitive disorders, as screened by medical doctors. Of the 843 patients eligible for the study, 72 declined to participate, which gives a participation rate of 91%. Thus, 771 adults with a medically confirmed diagnosis of HIV infection provided informed consent to participate in the study. After the informed consent was obtained, the study participants completed a paper version of the questionnaires. The study was approved by the local ethics commission. One person was excluded from the final dataset due to high percentage of missing answers. Table 1 describes sociodemographic and clinical characteristics for the final sample of 770 participants in detail.

**Table 1:** Sociodemographic and clinical variables in the studied sample (*N* = 770)

Variable	*N* (%)
Gender	
Male	599 (77.8%)
Female	171 (22.2%)
Age in years (*M* ± SD)	38.58 ± 10.31
Marital status	
In relationship	440 (57.1%)
Single	330 (42.9%)
Education	
Elementary	33 (4.3%)
Basic vocational	79 (10.3%)
Secondary	270 (35.1%)
University degree	388 (50.4%)
Employment	
Full employment	548 (71.2%)
Unemployment	101 (13.1%)
Retirement	24 (3.1%)
Sickness Allowance	97 (12.5%)
Financial status (from 1 = very low to 5 = very high)	2.50 ± 0.94
Sexual orientation	
Heterosexual	282 (36.6%)
Homosexual	413 (53.6%)
Bisexual	75 (9.7%)
Place of infection	
Home country	694 (90.1%)
Abroad	76 (9.9%)
Mode of infection	
Sex with men	525 (68.2%)
Sex with women	85 (11.0%)
Drugs	97 (12.6%)
Medical procedures	8 (1.0%)
Others	54 (7.0%)
HIV/AIDS status	
HIV+ only	629 (81.7%)
HIV/AIDS	140 (18.2%)
HIV infection duration in years (*M* ± SD)	8.07 ± 7.57
Antiretroviral treatment duration in years (*M* ± SD)	6.27 ± 5.86
CD4 count (*M* ± SD)	504.63 ± 238.65
Viremia	
Detectable	193 (25.1%)
Undetectable	518 (67.3%)
Don’t know	58 (7.5%)
Addiction	
Yes	117 (15.2%)
No	653 (84.8%)

*M* mean, *SD* standard deviation

### Measures

#### Personality dimensions

Personality was measured using the NEO-Five Factor Inventory (NEO-FFI) proposed by Costa and McCrae [35]. The NEO-FFI consists of 60 items (12 per trait), and participants respond to each item on a five-point scale from 0 (strongly disagree) to 4 (strongly agree). Higher summarised scores imply higher levels of each trait. The Cronbach’s alpha coefficients for the current study were .82 for neuroticism (N), .69 for extraversion (E), .61 for openness to experience (O), .71 for agreeableness (A) and .53 for conscientiousness (C). The Cronbach’s alpha obtained in the official adaptation of NEO-FFI [36] in the general Polish sample were .80 for neuroticism (N), .77 for extraversion (E), .68 for openness to experience (O), .68 for agreeableness (A) and .82 for conscientiousness (C).

#### Subjective well-being indicators

Subjective well-being was evaluated using the Satisfaction with Life Scale [SWLS; 37] together with the Positive and Negative Affects [PANAS-X; 38], according to the conceptualisation proposed by Diener. He defined subjective well-being as individual cognitive and affective evaluations of person’s own life [39]. The SWLS measures overall satisfaction with life. It is composed of five items on a seven-point scale ranging from 1 (strongly disagree) to 7 (strongly agree). Thus, a higher total score indicates higher level of life satisfaction. Cronbach’s alpha coefficient in the studied sample was .87. The affective component of subjective well-being describes an experience of longer-lasting emotional responses, including both positive and negative affects. Thus, 20 descriptions of feelings and emotions from the PANAS-X were used: 10 for positive affect (e.g. ‘proud’, ‘excited’) and 10 for negative affect (e.g. ‘depressed’, ‘stressed’). Participants rated their answers on a five-point response scale from 1 (not at all) to 5 (strongly). The Cronbach’s alpha coefficients obtained in this study were .86 for the positive affect scale and .91 for the negative affect scale.

## Data analysis

A comparison between the general population and PLWH on every Big Five personality dimension was made with the one-sample *t* test. Next, we used latent profile analysis to the identified types of people who are at the same time highly similar on personality traits within their group and highly dissimilar across the groups [40]. Analysis was performed on standardised values (*z*-scores), with reverse values for N to facilitate interpretation, which in such case should be understood as emotional stability [ES; see 5]. Models from one- to five-profile solutions were examined; as there have been only a few studies on clinical samples, the optimal solution could be different from the most popular in the healthy populations (i.e. three personality profiles).

To choose between competing models, we used a variety of indicators. For Akaike’s information criterion (AIC), Bayesian information criterion (BIC) and the sample-size adjusted BIC (SABIC), the lowest values indicate a model with the best fit [41]. The Vuong–Lo–Mendell–Rubin likelihood ratio test (VLMR) and the adjusted Lo–Mendell–Rubin likelihood ratio test (LMR) directly compare neighbouring *k* − 1 and *k* profile models; significant *p*-values suggest that the *k* profile model fits the data better than a model with one profile less [42]. Entropy is an index of classification accuracy, and values closer to 1 indicate better profile separation [43]. Finally, a size of the smallest profile is a practical criterion since a profile covering less than 5% of the sample may be hard to replicate. However, in clinical samples, even such small-size profiles may reflect rare but meaningful subgroups; thus the final decision on a number of profiles should be based on thorough inspection.

After establishing a number of profiles (here personality types), a bias-adjusted three-step procedure [43] was used for both testing their significant correlates (auxiliary variables; maximum likelihood method) and relationship with distal outcomes in terms of SWB dimensions (Bayesian hierarchical clustering method) [43]. Additionally, since this automatic procedure does not allow directly for control of correlates when examining the profile membership as a predictor of SWB, we repeated it manually as recommended by Asparouhov and Muthén [44]. Namely, after establishing profile membership in step 1, we specified the posterior profile membership probability as logistic function of the correlates in step 2; [44] and such values were used then in further analysis of relationship with distal outcomes. Thus, they can be interpreted as adjusted analysis controlled for the potential sociodemographic and clinical confounders. The analyses were performed by means of IBM SPSS Statistics version 25 [45], Mplus version 8.2 [46] and Latent Gold version 5.1.0.19007.

## Results

### Descriptive statistics and comparison with the general population on Big Five traits

Table 2 provides the basic descriptive statistics for our sample and results of comparison with the general population. The population data are taken from an official adaptation and standardisation sample on NEO-FFI [36; *N* = 2041, mean age = 27.51 ± 13.25 years, 52% women, 11% university degree]. PLWH are on average lower on four out of five dimensions: E, O, A and C, and the same as general population on N.

**Table 2:** Descriptive statistics and one-sample *t* test for comparison with population means on big five dimensions

Variables	Sample *N* = 770					Population *N* = 2041		*t*	*p*	Cohen’s *d*
	M	SD	Min–max	Skewness	Kurtosis	*M*	SD			
Personality										
N	22.44	8.78	0–46	− .10	− .42	22.79	7.87	− 1.10	ns	0.04
E	23.47	5.73	8–39	− .14	− .02	27.79	6.86	− 20.93	< .001	0.75
O	25.34	5.92	10–41	.27	− .40	27.80	6.31	− 11.54	< .001	0.42
A	27.93	6.47	8–44	− .03	− .45	28.68	5.76	− 3.24	.001	0.12
C	26.52	4.99	0–41	− .39	2.26	29.40	7.25	− 16.02	< .001	0.58
Subjective wellbeing										
SWL	19.13	6.43	5–35	− .02	− .64					
PA	3.32	0.72	1.2–5	− .18	− .34					
NA	2.23	0.90	1–5	.67	− .24					

*N* neuroticism, *E* extraversion, *O* openness to experience, *A* agreeableness, *C* conscientiousness, *SWL* satisfaction with life, *PA* positive affect, *NA* negative affect

Due to these differences, latent profile analysis was carried out twice: once on data standardised by sample means, and then on data standardised by population means. The first analysis results allow for interpretation in the sample-relative terms only, whereas in the second case they can be related to the population-specific values, and hence treated as more absolute. For example, a higher profile obtained in the first analysis means only higher for a given sample, whereas in the second case, it is higher ‘in general’, i.e. also in relation to the averages for the general population.

### Personality profiles: Sample-standardised personality dimensions

**Fig. 1:**
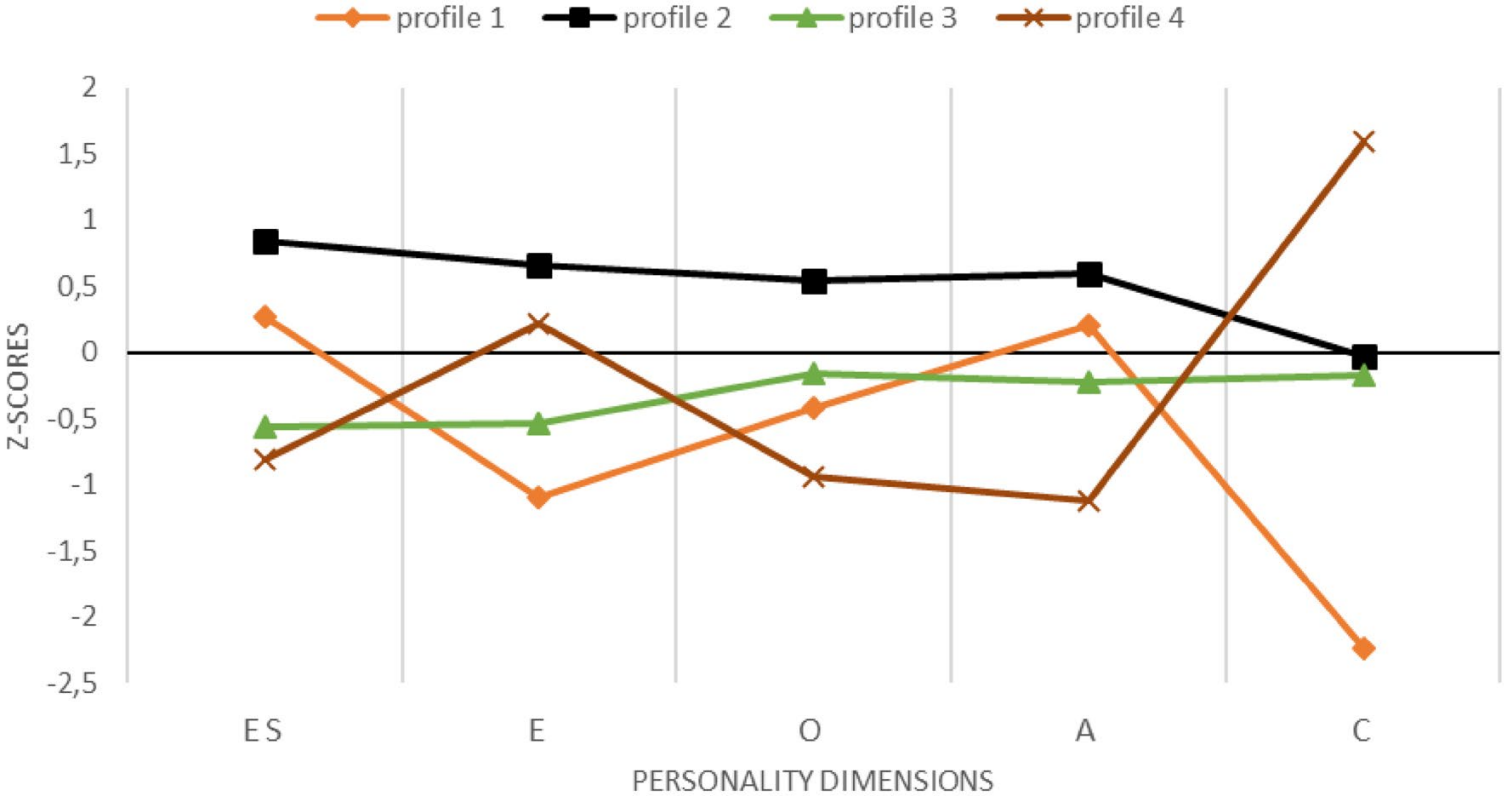
Results of latent profile analysis on the sample-based standardisation: four profiles of Big Five personality dimensions. *ES* emotional stability (reverse scores of Neuroticism), *E* extraversion, *O* openness to experiences, *A* agreeableness, *C* conscientiousness. Profile 1—undercontrolled type; profile 2—resilient type; profile 3—average type; profile 4—overcontrolled type

The results of latent profile analysis are presented in Table 3. The four-profile solution is better fitted to the data than the assumed three-profile model: namely, the former has lower values of AIC, BIC and SABIC, higher entropy and significant values of LMR and VLMR likelihood ratio tests. For the latter the values are insignificant when comparing models with four and five profiles that additionally points to the 4-profile solution. The main difference between three- and four-profile models arises due to extraction of a profile with the lowest C (see Fig. 1). Thus, profile 1 (6% of the sample, 47 participants) may be considered equivalent to undercontrollers, but the most frequent characteristics of this type include being low on O and A, which is not the case here. PLWH in this profile are very low on C (below two standard deviations) and rather introvert. Profile 2 (39%, 300 participants) resembles a resilient profile, with all the dimensions being above the sample average, with C being the only exception. Profile 3 (42%, 320 participants) is an average profile but with a tendency to be rather below the sample mean. Profile 4 (13%, 103 participants) can be identified as overcontrollers: high on C, low on ES but—which is untypical for this profile—not low on E.

**Table 3:** Summary of model selection indices of latent profile analysis

Model	BIC	AIC	SABIC	No of parameters	Entropy	LMR		VLMR		Smallest class	
						Value	*p*	Value	*p*	% of *N*	frequency
Sample-based standarisation											
1-Class	10,992	10,946	10,961	10							
2-Class	10,725	10,650	10,674	16	.61	300.53	.05	81.82	.05	47.99	370
3-Class	10,555	10,453	10,485	22	.67	204.30	< .001	− 51.73	< .001	18.57	143
4-Class	10,318	10,318	10,359	28	.76	143.70	< .001	2.04	< .001	6.10	47
5-Class	10,417	10,259	10,309	34	.75	68.67	ns	77.166	ns	7.01	55
Population-based standarisation											
1-Class	10,382	10,335	10,350	10							
2-Class	10,110	10,036	10,059	16	.61	303.88	.04	76.25	.04	49.61	382
3-Class	9946	9843	9876	22	.66	199.51	< .001	− 41.74	< .001	18.57	143
4-Class	9829	9699	9740	28	.76	153.11	.002	7.01	.001	6.10	47
5-Class	9799	9641	9691	34	.75	68.18	ns	111.14	ns	6.36	49

*BIC* Bayesian information criterion, *AIC* Akaike’s information criterion, *SABIC* sample-size adjusted BIC, *LMR* Lo–Mendell–Rubin likelihood ratio test, *VLMR* Vuong–Lo–Mendell–Rubin likelihood ratio test

### Personality profiles: Population-standardised personality dimensions

**Fig. 2:**
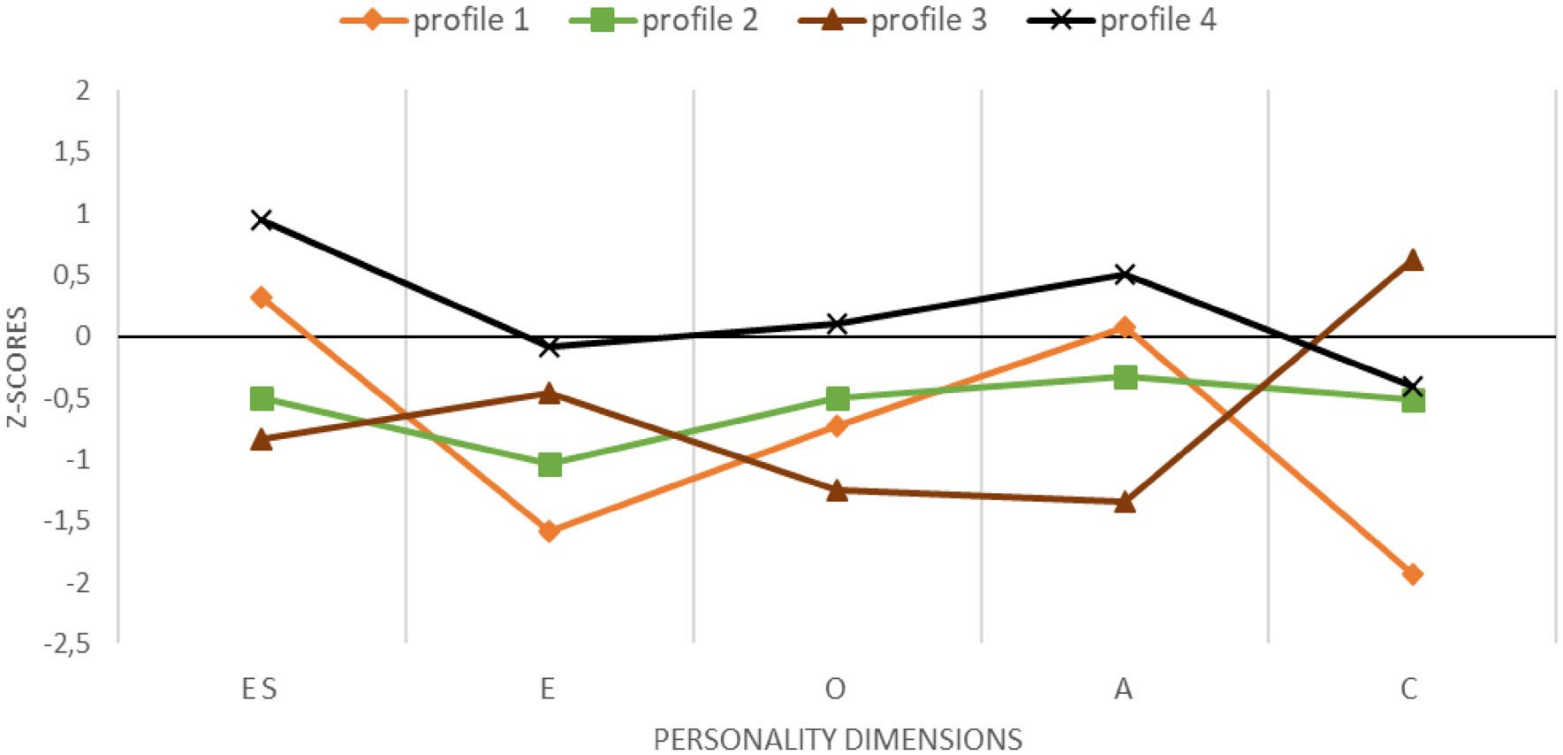
Results of latent profile analysis on the population-based standardisation: four profiles of Big Five personality dimensions. *ES* emotional stability (reverse scores of Neuroticism), *E* extraversion, *O* openness to experiences, *A* Agreeableness, *C* conscientiousness

Congruently, a solution with four profiles can be regarded as optimal (see Table 3, bottom panel). The obtained profiles are highly similar to those for the previous analysis in terms of counts, shape and posteriors probabilities of belonging to a given profile (i.e. correlation from .98 to 1 indicates almost perfect overlap). The interpretation should be attuned mainly for the resilient profile; now it is more like an average profile albeit with higher than typical ES (see profile 2 in Fig. 1 and profile 4 in Fig. 2). The previous average profile now turns into low profile (i.e. coherently below average; profile 3 in Fig. 1 and profile 2 in Fig. 2) and the differences in C for the other two profiles are less pronounced. Nevertheless, due to the similarities in the results of both analyses, in particular, the very high correlations between the probabilities of being a member of the corresponding profiles, further analyses will be based on the sample-standardised solution only (Fig. 1).

### Sociodemographic and clinical correlates of profiles

The sociodemographic and clinical variables presented in Table 1 were included in the analysis. We found no significant relationship with a profile membership in terms of gender, age, education, employment, self-assessed financial status, sexual orientation or current romantic relationship status. Concerning clinical variables, the personality profiles also did not differ significantly in terms of CD4 count, self-declared viremia, AIDS stage or the presence of addiction. The only exceptions were mode and place of HIV infection. Specifically, a different pattern was observed for undercontrollers: less-frequent infection due to sex with men (47 vs. 70% average for all the other profiles), more-frequent infection due to sex with women (28 vs. 11%), and more frequently reported ‘other sources of infection’ (13 vs. 7%) as well as being infected outside the country (none in undercontrollers in comparison to approximately 10% in all the other profiles). Thus, underconntrollers differ significantly from other personality profiles in terms of both mode and place of infection, but they did not differ in terms of sociodemographic variables and indicators of disease control and progression.

Additionally, since duration of HIV infection was strongly correlated with years of ART (.82, *p* < .001), we repeated the analysis with only one of these variables at a time. It did not change the general pattern of their similar values across profiles.

### Profiles’ membership and SWB

As presented in Table 4, SWB differs across personality profiles. The highest SWB is observed among members of resilient profile: they differ significantly on each SWB dimension from all the others profiles, for which the picture is unclear (see note on pairwise comparisons). Namely, overcontrollers and undercontrollers can be almost equally regarded as the second best, whereas average profile is related to the lowest SWB. When this analysis was adjusted for relationship between the profile membership and correlates, the patterns of results remained the same (bottom panel of table).

**Table 4:** Relationship between profiles and subjective well-being (means)—overall Wald test

Subjective wellbeing	Profile 1	Profile 2	Profile 3	Profile 4	Wald	
	Undercontrollers *n*_1_ = 47	Resilient *n*_2_ = 300	Average *n*_3_ = 320	Overcontrollers *n*_4_ = 103	Value	*p*
	*M*	*M*	*M*	*M*		
Without adjustment for profile membership correlates^a^						
Satisfaction with life	17.28	23.72	15.33	18.71	203.79	< .001
Positive affect	2.89	3.77	2.96	3.35	163.10	< .001
Negative affect	2.15	1.64	2.68	2.53	185.43	< .001
With adjustment for profile membership correlates^b^						
Satisfaction with life	17.29	23.79	15.42	18.69	202.21	< .001
Positive affect	2.89	3.77	2.96	3.34	163.98	< .001
Negative affect	2.17	1.63	2.66	2.54	182.78	< .001

^a^All the pairwise comparisons between profiles significant at least at *p* < .05. Exceptions are: profile 1 versus profile 3 (Wald = 3.38. df = 1. *p* = .07) and profile 1 versus profile 4 (Wald = 1.48. df = 1. *p* = .22) for Satisfaction with life; profile 1 versus profile 3 (Wald = 0.22. df = 1. *p* = .64). for Positive affect; profile 3 versus profile 4 (Wald = 1.41. df = 1. *p* = .24) for Negative affect

^b^All the pairwise comparisons between profiles significant at least at *p* < .05. Exceptions are: profile 1 versus profile 3 (Wald = 2.99. df = 1. *p* =  .08) and profile 1 versus profile 4 (Wald = 1.38. df = 1. *p* = .24) for Satisfaction with life; profile 1 versus profile 3 (Wald = 0.45. df = 1. *p* = .50) for Positive affect; profile 3 versus profile 4 (Wald = 1.03. df = 1. *p* = .31) for Negative affect

## Discussion

The results of our study showed significant differences on four personality traits between HIV-infected participants and the general population—the observed effect size was strong for E, medium for C and O and small for A, with no effect for N. This finding is an interesting contribution to the HIV literature, as no such comparison has been conducted to date. Thus, our result may add to the long but less-conclusive debate if a specific personality profile of PLWH exists in comparison with the general population. Specifically, the authors argue whether certain personality dimensions may be linked to premorbid mood disorders and associated risky behaviours that act as potential pathways to HIV infection [34, 47] or that changes in some personality dimensions may be the result of ongoing adaptation to potentially fatal and still very stigmatising disease [22, 48]. However, similar to the studies mentioned above, we did not control for the causality with respect to the link between personality and socio-medical variables, and without any longitudinal studies on this topic to date, the possibility to interpret personality-risk associations among HIV-positive compared with HIV-negative individuals is very limited [48].

Thus, it seems that the major result obtained in this study deals with identifying personality profiles among PLWH, which also have not yet been published in the HIV/AIDS literature. Particularly, we failed to identify the most frequently reported three profiles [3, 4], but we managed to extract a four-profile model on both standardisations (i.e. sample-specific and population level). Namely, the resilient type (emotionally stable, extravert, open to experience and agreeable) and, similar to the latter, albeit with a significantly lower profile, the average type both together cover almost 81% of our sample. In contrast, only 13% of participants could be classified as overcontrollers (i.e. high on C, low on ES, but—which is untypical for such profile—not low on E). Finally, only 6% of our sample could be identified as undercontrollers; however, in the literature, the most frequently reported profile for this type is low also on A, which is not the case here (i.e. in our sample, PLWH representing this type are very low only on C and, additionally, low on E, which is also not typical).

It should be noted that a similar four-profile model was obtained in other studies, albeit in non-clinical settings [5]. Even if our sample cannot be considered as randomly coming from the general population in terms of personality dimensions, it remains internally heterogeneous with respect to personality traits. Thus, our results may shed new light on two inconclusive issues of personality characteristics among PLWH. Specifically, we observed that participants in almost all personality profiles did not differ in terms of all studied socio-medical correlates. Only the undercontrolled type was different with respect to the mode of HIV infection (i.e. less-frequent infection due to sex with men, more frequent infection due to sex with women and more frequently reported ‘other sources of infection’, which is a category separate from these related to drug abusing or hospital infections) as well as being infected outside the country. This result is intriguing, as it suggests that personality is unrelated especially to HIV-related clinical variables, which is opposed to several studies conducted so far [30, 31, 48]. However, it should be noted that these studies were internally inconsistent with each other, highlighting sometimes contradictory findings on the role of the same trait with various medical outcomes of PLWH (e.g. extraversion, neuroticism, openness or conscientiousness). Thus, it seems that personality types are much more nuanced, though an insignificant picture of this association was compared with simply basing on single and isolated traits. Alternatively, the finding connected to different behaviours of undercontrolled type with regard to the mode and place of HIV infection may be interpreted in the light of classic theory that the relation between personality and health is explained by health behaviours, which mediates its association [49]. In a more recent review, Shuper et al. [50] underlined that personality has a strong effect on risky sexual behaviours among PLWH. One should remember that what characterised the undercontrolled type mostly was the lowest level of conscientiousness. Several meta-analytic reviews documented a positive link between this trait and health-promoting behaviours in the general population [51], including studies conducted with PLWH [52].

The second ambiguous topic deals with the link between personality and well-being for PLWH [31, 32]. In our study, the highest SWB was observed among members of the resilient profile, that differed significantly on each SWB dimension from all the others profiles, for which the picture was less clear. Namely, overcontrollers and undercontrollers were almost equally regarded as the second best in the level of SWB, whereas the average profile consists of PLWH with the worst SWB. The highest SWB in the resilient profile is in line with other studies documenting many positive psychosocial outcomes among people representing this type, yet conducted in non-clinical settings only [11]. However, in our sample, the resilient profile was in fact an average profile in terms of population means, which may serve as an explanation why we noted the highest SWB for this profile. Although causality is not proven here, an intriguing finding is that a typical personality for a given society is related to better SWB, even for PLWH. In other words, being more like an average on each personality dimension played the major role in SWB level among participants, neither specific personality traits nor its constellation [e.g. conscientiousness, 48 or extraversion, 32].

It should be underlined that standardisation may matter for interpretation of the results, especially in the context of clinical samples, which may differ on personality traits from the general population [for PLWH, 33] and regarding the explorative nature of LPA, where extracted profiles may be strongly sample-related [39]. We did not know of any other study that considered this possible source of bias. We have attempted to overcome it by referring our results to the personality profiles most frequently reproduced thus far [9] and to population-based standardisation.

In addition, overcontrollers and undercontrollers, although representing reverse profiles, were very similar in terms of SWB. Nevertheless, it has to be stressed that these profiles do not entirely resemble those reported in the literature [4]. The main differences regard the levels of E and A. Thus, there is a need to conduct further research on these personality types among PLWH. The same applies to the last, the sample-average type, but below average in terms of population means. This group consisted of 42% of the sample; however, hardly anything specific can be said about this group without falling into speculative remarks.

## Strengths and limitations

This study has several strengths, including a large size of clinical sample, two methods of standardisation (i.e. sample and population) and the person-oriented approach to personality. However, a few limitations should also be noted. Firstly, the cross-sectional design precludes causal interpretations. Secondly, our sample was composed mostly of highly functioning PLWH, with unequal distribution of gender and sexual orientation (mostly homosexual men and heterosexual women). However, this specific gender and sexual distribution reflects the current distribution of these variables among PLWH in Poland [53] and in most European countries [54]. Also, it should be mentioned that we obtained relatively low reliability of the NEO-FFI subscales, which could be related to the sample specificity. Finally, due to ethical and legal issues related to data protection (i.e. third-party access to medical records), we based our analysis only on self-reported clinical variables.

## Conclusion

Identifying personality types in clinical settings enables more comprehensive understanding of interrelations between personality and health [15, 16]. However, additional studies are required to determine whether these types of personality are universal as well as why some studies failed to extract them or obtain types that do not entirely resemble those reported in the literature [8], which was also the case of our study. Concerning PLWH, the typological approach to the study of personality may clarify many ambiguous results devoted to the role of personality traits across various aspects of functioning of PLWH, including the issue of well-being of these patients.
